# The impact of a vegetarian diet on chronic kidney disease (CKD) progression – a systematic review

**DOI:** 10.1186/s12882-023-03233-y

**Published:** 2023-06-12

**Authors:** Łukasz Świątek, Jan Jeske, Miłosz Miedziaszczyk, Ilona Idasiak-Piechocka

**Affiliations:** Department of Nephrology, Transplantology and Internal Medicine, Poznan University of Medical Sciences, Poznan, Poland

**Keywords:** Chronic kidney disease, eGFR, Vegetarian diet, Vegetarianism

## Abstract

**Background:**

A vegetarian diet is a popular alternative to the casual diet - it is considered healthy, and was proven to positively affect cardiovascular health. The Chronic Kidney Disease (CKD) progression is a major issue in the healthcare system, and constitutes a leading cause of death for 1.5% of the global population. The objective of this systematic review was to investigate the potential impact of a vegetarian diet on kidney function in CKD patients.

**Method:**

Our systematic review focused on randomized controlled trials (RCTs) which compared the effects of a vegetarian diet (experimental) and a standard omnivore diet (comparator) in terms of the estimated glomerular filtration rate (eGFR) in CKD patients. Inclusion criteria were based on PICO elements, with two researchers involved in browsing the Cochrane and Pubmed search engines. The investigation was performed using the PRISMA 2020 Checklist and PRISMA 2020 flow diagram. The search terms included: ‘vegetarian diet’ AND ‘nephropathy’, ‘eGFR’, ‘albuminuria’, ‘chronic kidney disease’. Bias assessment was performed using RoB 2 tool to determine the validity of the data collected from studies.

**Results:**

Four RCTs with a total of 346 participants were included in the presented systematic review. Two largest RCTs reported an increase in eGFR following a change to a vegetarian diet (p = 0.01 and p = 0.001). Another two found no significant differences between the experimental and control groups, also these trials were associated with a high risk of bias in terms of missing data outcome and the randomization process.

**Conclusions:**

The findings collected in this systematic review suggest that a vegetarian diet improves renal filtration function in CKD patients. Therefore, it seems essential to conduct further research involving the impact of the diet on the progression of CKD.

**Supplementary Information:**

The online version contains supplementary material available at 10.1186/s12882-023-03233-y.

## Background

According to the National Health and Nutrition Examination Survey (NHANES), Chronic Kidney Disease (CKD) affects approximately 15% of adults in the United States, amounting to over 30 million people [1]. In fact, patients with a glomerular filtration rate (GFR) below 60 mL/min /1.73 m² present a 57% higher possibility of cardiovascular mortality; in such cases the risk of suffering stroke is also 7% higher for every 10 mL/min/1.73 m² decrease in GFR [2].

Vegetarian and vegan diets, along with their various types, have become increasingly popular worldwide. The term “vegetarian diet” is most commonly associated with a lacto-ovo vegetarian diet, in which fruits and vegetables are consumed, as well as dairy products (such as milk and cheese), eggs and honey. Another type of vegetarian diet is pescetarianism, which also includes fish. In contrast, in a vegan diet, only the consumption of fruits and vegetables is allowed [3, 4]. Vegetarian diet types are summarized in Table 1.

**Table 1:** Vegetarian diet models [3, 4]

Diet model	Products excluded from the diet
Pescetarianism	meat
Ovo-lacto-vegetarianism	meat, fish
Ovo-vegetarianism	meat, fish, milk
Lacto-vegetarianism	meat, fish, eggs
Veganism	meat, fish, eggs, milk, honey

**Fig. 1:**
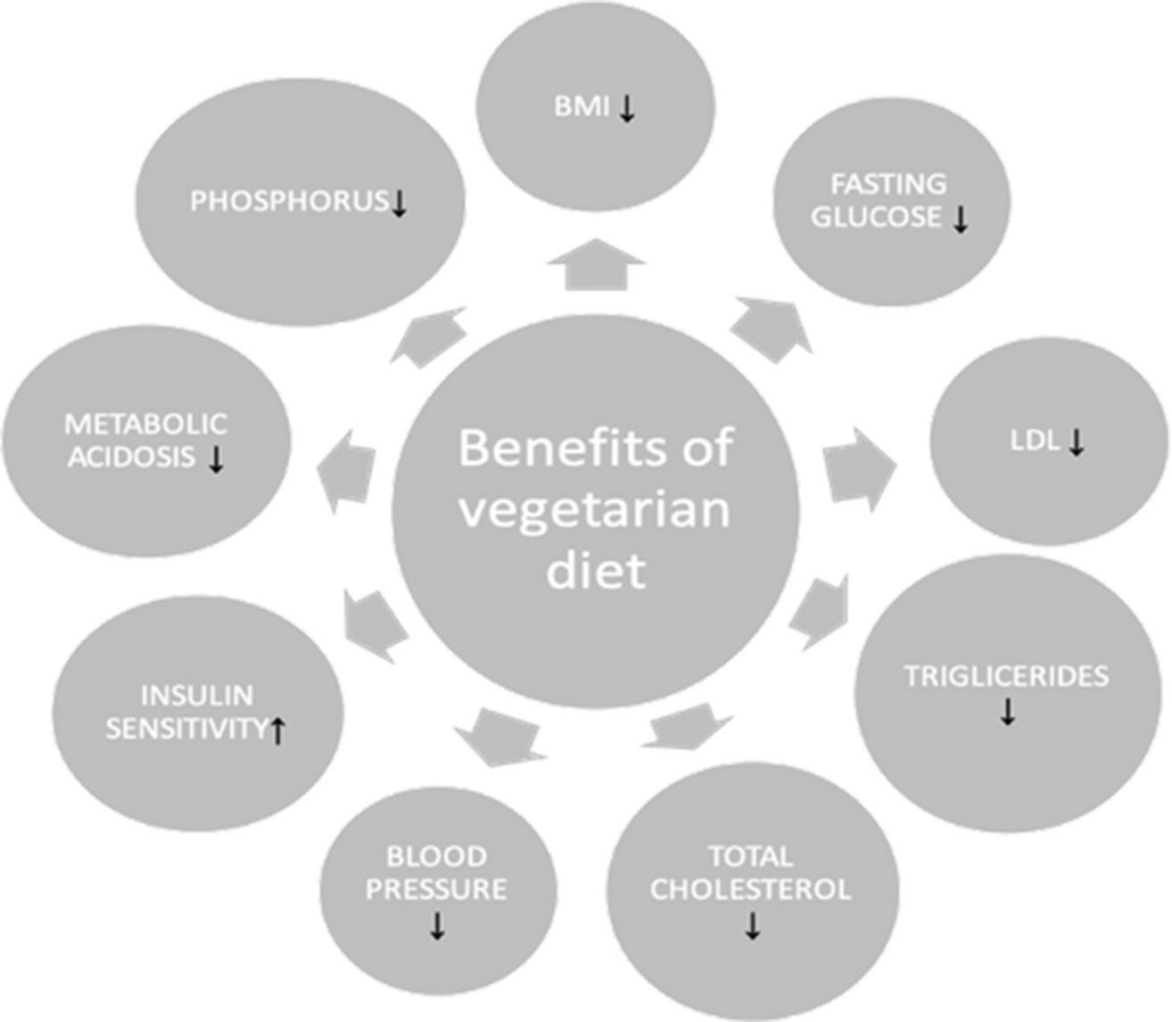
The potential benefits for patients changing to a vegetarian diet

Vegetarians and vegans show lower levels of numerous common risk factors, such as BMI (-1.72 kg/m2), fasting glucose (-6.38 mg/dL), LDL-cholesterol (-22.87 mg/dL) and triglycerides levels (-9.35 mg/dL) in comparison to individuals consuming meat [5]. Another meta-analysis shows that, by means of dietary changes, total cholesterol (TC) and low-density lipoprotein cholesterol (LDL-cholesterol) levels may be reduced by up to 15% in the case of a lacto-ovo vegetarian diet; up to 25% in a vegan diet, and as much as 35% in terms of a vegetarian diet when certain nutrients, such as fiber, soy and nuts are added [6]. Moreover, according to another study, vegetarians presented a decrease of 9.1 mmHg in systolic BP and of 5.8mmHg in diastolic BP compared to non-vegetarians [7]. Similar findings were observed in vegans who showed a decrease of 6.8mmHg and 6.9mmHg in systolic and diastolic BP, respectively [7]. Vegetarians also present a lower fasting glucose level and a higher insulin sensitivity than omnivores, with 12% lower β-cell function, whereas the calculated Homeostatic Model Assessment – Insulin Resistance (HOMA-IR) for vegetarians was 1.10 compared to 1.56 for omnivores (p = 0 = 0.001) [8]. It is worth bearing in mind that a vegetarian diet is beneficial for phosphorus homeostasis, as confirmed by the positive effect of plant-based protein source, which significantly decreased fibroblast growth factor 23 (FGF23) levels along with serum phosphorus levels [9]. Additionally, a high consumption of grain, fruits and vegetables is associated with a reduction in metabolic acidosis which might be beneficial for CKD patients [10]. A graphic representation of the potential health effects of a vegetarian diet are presented in Fig. 1.

One of the nutritional strategies applied in the course of CKD is the protein restriction therapy, i.e. either a low protein diet (LPD) (0.6-0.8 g/kg/day), or a very low protein diet (VLPD) (0.3-0.4 g/kg/day) which is additionally supplemented with ketoanalogues (KAs) [11], [12]. There are various studies showing that the introduction of some nutrition modifications may delay the development of CKD. Therefore, the presented systematic review aimed to investigate the potential positive effects of a vegetarian diet on kidney function in patients with CKD.

## Method and materials

**Fig. 2:**
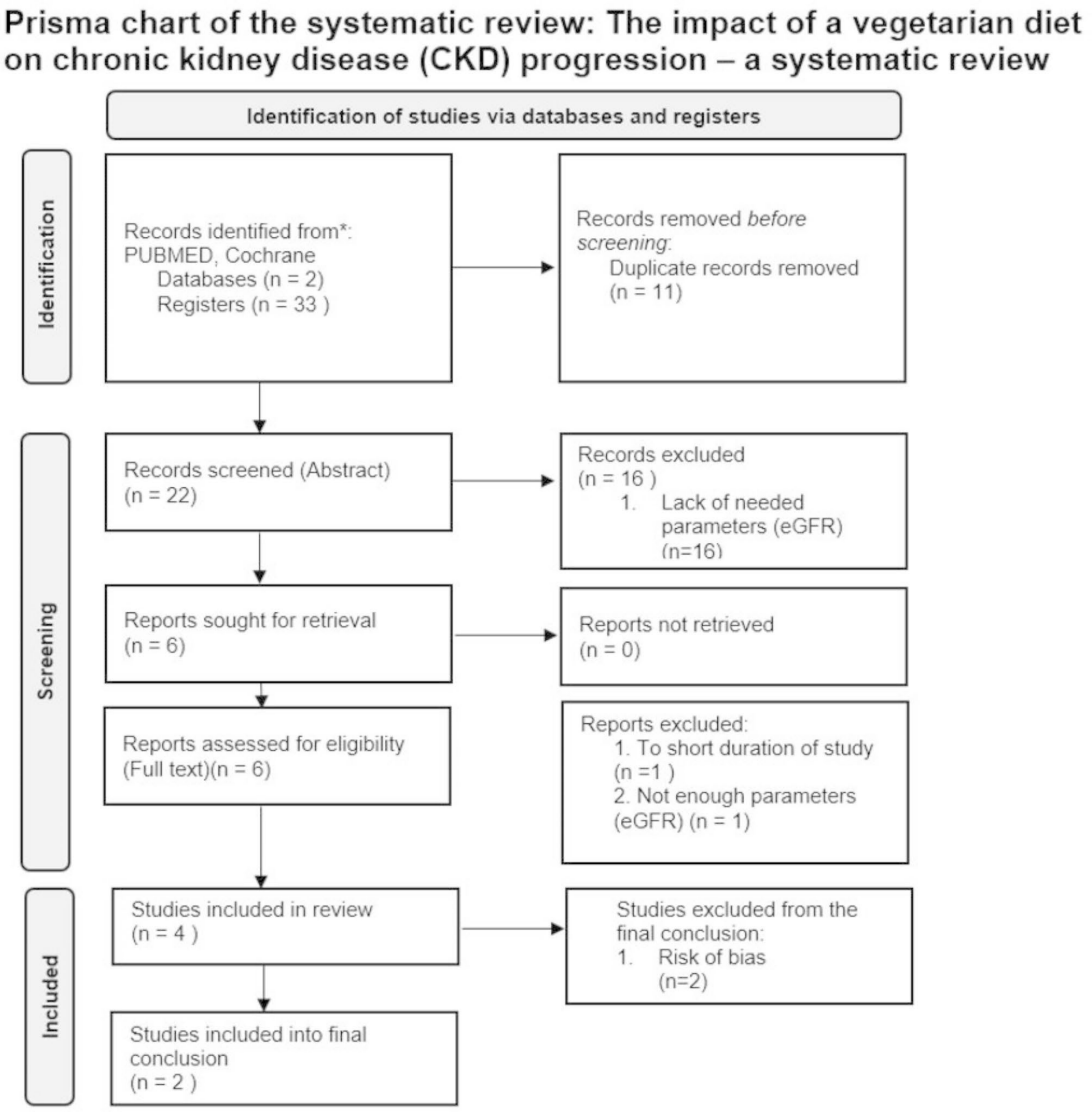
Prisma chart of the systematic review

The research strategy was based on search terms. The investigation was performed according to the PRISMA 2020 Checklist and PRISMA 2020 flow diagram which is presented in Fig. 2 [13]. The selected search engines for this research were Pubmed and Cochrane, and the selected terms for this research comprised: ‘vegetarian diet’ AND ‘nephropathy’, ‘eGFR’, ‘albuminuria’, ‘chronic kidney disease’. The search included randomized controlled trials and non-randomized controlled trials; however, only in papers published in English. Due to a small number of studies, the criterion of year was not applied.

### Inclusion criteria

Inclusion criteria were designed using the PICO elements: The population investigated was the CKD patients population (Population; P). Intervention (I) involved the introduction of the plant-based diet, including subgroups of a vegetarian and vegan diet. The study groups were expected to be compared to a control group - the omnivore (standard) diet (Comparison; C). The final outcomes should be measured by comparing the initial eGFR and final eGFR at the end of the study (Outcomes; O). The exclusion criteria comprised: all studies with a duration shorter than 4 weeks and research in a language other than English. Further criteria were not applied due to a lack of studies. Two researchers were involved in the selection process. They worked independently, their views regarding the studies were subsequently confronted and discussed. Moreover, authors developed a data extraction tool. The variables extracted from the studies included: diet type, study duration, year, group size, the initial and final eGFR, as well as P value.

### Data collection process

At the identification stage, 33 records were included from 2 databases, i.e. Cochrane and Pubmed, and after removing duplicates − 22 records were screened. 16 records were excluded due to inadequate, or lack of data. The remaining six full papers were searched. Four RCTS qualified for inclusion in the review. A summary of the screening process is presented in Fig. 2. Two papers assessed as eligible for review, were removed, since they did not meet the inclusion criteria.

The paper comparing the effects of consuming a meat meal and a meat free meal by Preiss et al. [14] was excluded due to the short duration of the study (several hours). Although the study compared two diets, it did not provide the long-term effects of the diet on eGFR.

Another study which failed to meet the eligibility criteria was the paper comparing a vegetarian and omnivore diet in terms of protein source and phosphate homeostasis. The study comprised CKD patients in stage 3–4. The initial mean eGFR was known; however, final eGFR was not measured. Moreover, the duration of the study was 7 days, which was considered too short to meet the inclusion criteria [15].

### Risk of bias assessment method

**Fig. 3:**
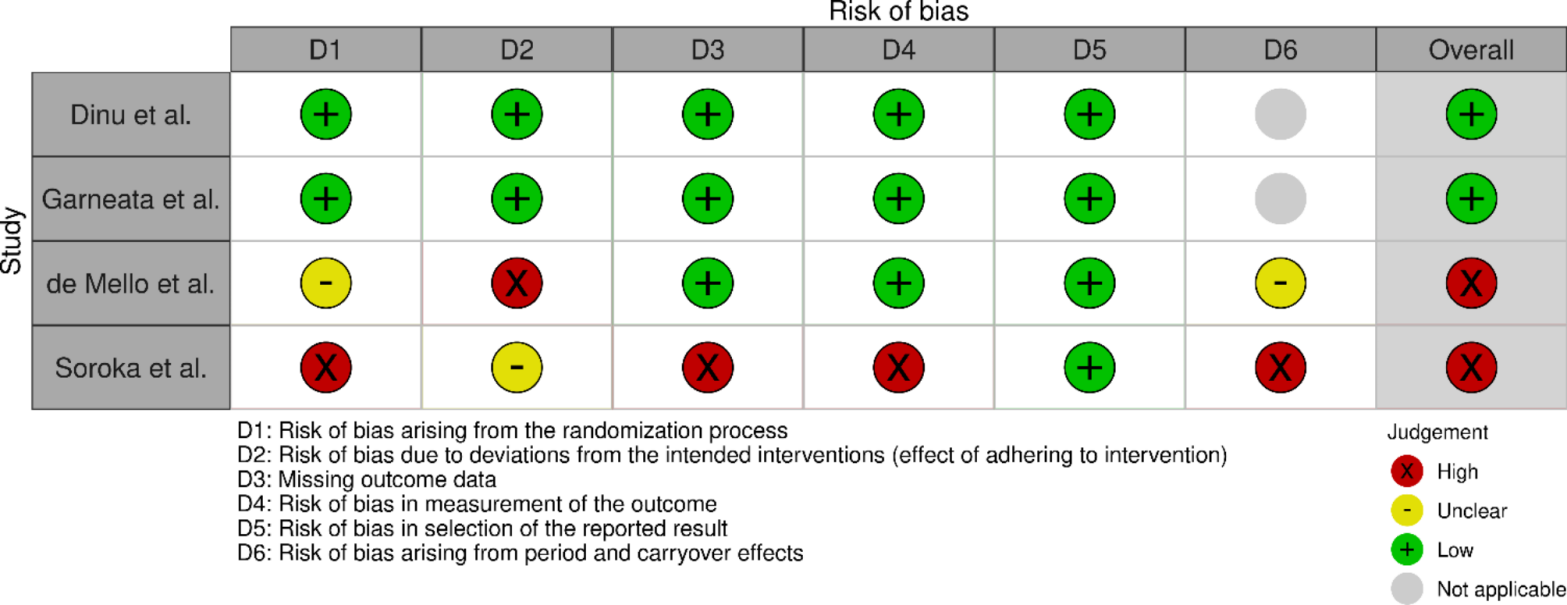
Bias assessment “traffic light” plots of the domain-level assessment for each individual outcome

The bias assessment was conducted using the RoB 2 tool for each individual study, by addressing signaling questions in order to determine the risk of bias [16]. Two researchers were involved in this process. The sources obtained for bias assessment included articles and study protocols, if available. The written consensus of authors discussion in the form of complete templates are presented in supplement 1–4. The effect of bias assessment is presented as a forest plot in Fig. 3. The risk of bias will be used to determine the validity of the data extracted from the studies that met the inclusion criteria. This bias analysis will be essential for drawing conclusions from the accessed data by grading the risk of bias. Studies graded “low” will account for the final conclusion.

## Results

### The effects of a vegetarian diet on kidney function

Two studies, by Dinu et al. [17] and Garneata et al. [18] confirmed a significant positive impact of a vegetarian diet on eGFR in the experimental group with the p value of 0.001 and 0.01, respectively. Nevertheless, there were also two studies which did not find either a positive, or a negative impact of a vegetarian diet on GFR. Both of these studies showed that there was no significant difference in eGFR between the experimental and control group [19, 20]. The summary results for each study are presented in Table 2.

**Table 2:** eGFR results of studies comparing different diet types

Diet Type	Duration(year)	Size group	The initial eGFR mL/min/1.73m^2^	The final eGFR mL/min/1.73m^2^	P value	Reference
**Lacto**-**ovo**‐**vegetarian diet (VD), compared to a Mediterranean diet (MD)**	3 months 2021	54 VD 53 MD	96.5 ± 8.8 VD 97.0 ± 11.5 MD	99.9 ± 9.2 VD 95.7 ± 10.2 MD	0.001	(17)
**Ketoanalogue-supplemented vegetarian very low–protein diet (KD) vs. conventional low–protein diet (LPD)**	3 months 2016	104 KD 103 LPD	18.0 ± 11.9 KD 17.9 ± 13.0 LPD	15.1 ± 10.9 KD 10.8 ± 8.3 LPD	0.01	(18)
**Usual diet (UD) vs. chicken (CD) vs.** **lactovegetarian low-protein diet (LPD)**	4 weeks 2006	17 patients changing diets every 4 weeks	81.8 ± 22.2	81.8 ± 22.2 UD 83.3 ± 26.1 CD 81.9 ± 25.3 LPD	not significant	(20)
**Vegetarian** **low-protein diet (VPD) and an animal-based low-protein diet (APD)**	6 months 1998	15 patients changing diet after 6 months	28.8 ± 13.3	28.1 ± 3.4 VPD 29.6 ± 3.8 APD	not significant	(19)

A significant difference was observed between a lacto-ovo‐vegetarian diet (VD) and the Mediterranean diet (MD) with the mean difference of 4.2 mL/min/1.73 m² (p < 0.001) in the final eGFR. There was an 3.4 mL/min/1.73 m² increase between the initial and final eGFR in a VD group which indicated a positive impact of the diet on eGFR [17]. In the study involving a ketoanalogue-supplemented vegetarian very low protein diet (KD), a significant difference was found between the two diet types. A mean difference was observed between the final eGFR of 4.3 mL/min/1.73 m² (p < 0.01) of KD and a conventional low protein diet (LPD). Furthermore, a decrease of 2.9 mL/min/1.73 m² was reported in KD. However, it is crucial to note that the reduction in eGFR was lower in KD compared to the decrease in LPD (7.1 mL/min/1.73 m²). The other two studies presented no significant differences in the final eGFR between the study groups, thus, the changes between the groups were not analyzed [19, 20].

### Other renal parameters

In one study, a significant difference was found (p = 0.001) in creatinine level between a vegetarian 0.72 g/dL (0.69-0.74 g/dL) group and the Mediterranean diet 0.76 g/dL (0.74‐0.79 g/dL) group [17]. In addition, at the end of the study, there was a considerable change in the serum urea concentration between the groups consuming a ketoanalogue-supplemented vegetarian very low–protein diet (KD) – 120 mg/dL, and a low–protein diet (LPD) – 226 mg/dL [18]. Moreover, the rate of urinary albumin exertion (UAER) was visibly lower in the LPD group (229.3ug/min)^3^ in comparison to the UD (312.8ug/min)^3^ and CD (269.4ug/min)^3^ (P < 0.001) [20]. A significant reduction in serum phosphates was found in favor of the KD group 4.4 mg/dl (4.3–4.5) vs. LPD 6.2 mg/dl (5.8–6.5) [18].

### Bias assessment analysis

The presented systematic review involved two randomized cross-over trials and two randomized parallel-group trials. The risk of bias assessment of a study by Dinu et al. [17] was estimated using the study protocol [21]. This study demonstrated a low risk of bias in all domains of ROB2 tool. As the researchers were aware of the randomization process, a statistician was asked to perform it. Furthermore, allocation concealment was ensured, as well as blinding of data assessors, no missing data was present, and an appropriate data analysis was employed [17]. Another study presenting low risk of overall bias was conducted by Garneata et al. [18]. In fact, it was the largest study included in the review. It showed good randomization and allocation, and there were no significant baseline differences between the groups. Moreover, although it was an open-label study, it scored “low” in the “effect of adhering to intervention” domain. In spite of the fact that 7 patients discontinued the study, the risk of missing the outcome remained “low”, due to an insignificant change in the number of participants who finished the study. There was no measuring bias, and an appropriate analysis was also applied to determine the outcome [18].

The abovementioned two randomized parallel-group trials presented a low overall risk of bias, and thus are eligible to draw conclusions.

The study performed by de Mello et al. in 2006 [20] presented an overall high risk of bias. This crossover randomized trial showed certain issues with addressing the method of randomization, no 1:1 ratio in the first sequence was ensured and there was no data regarding the baseline differences between the groups in the first sequence. Additionally, although the study did not provide the number of participants is each sequence, it included the washout period. Nevertheless, there was a high risk of bias in D2 domain (effect of adhering to intervention), since no analysis of adhering to the intervention was performed [20].

Another crossover randomized trial by Soroka et al. in 1998 [19] which also presented a high risk of biased. A key issue was uneven patient allocation in the first sequences, where the ratio was 2:1, hence, indicating poor randomization quality. Moreover, the time for carry-over effects to disappear was insufficient and the patients switched to another diet as soon as the previous one was completed. In fact, a huge percentage of patients withdrew from the study. Just 9 out of 15 finished the study, which affected domains D2 and D3 (missing data outcome). However, there was a low risk of bias in the measurement of the outcome domain and the reported result. These two individually randomized cross-over studies demonstrated a poor quality assessment bias [19]. The bias assessment for each trial is summarized in Fig. 3.

## Discussion

Four RCTs with a total of 346 participants were included in this systematic review. In two largest RCTs, eGFR was reported to increase following a change to a vegetarian diet (P = 0.01 and P = 0.001). Another two reported no significant differences between the experimental and control groups, and these were associated with a high risk of bias in terms of missing data outcome, or the randomization process. The clinical application of a vegetarian diet in CKD prevention may be compared to a low-protein conventional diet (0.6-0.8 g/kg/day of proteins), due to its naturally low protein character [17] and positive impact on eGFR [17, 18] along with a decreased urinary albumin exertion [20]. In fact, the implemented bias assessment might discredit the aforementioned studies, due to the change in standards over the last 20 years. Therefore, introducing a time criterion for the trials may be the most appropriate solution. Moreover, the study groups in both trials were relatively small, involving no more than 17 patients, which also could have affected the final results. A study strictly comparing the difference between a low protein vegetarian diet and a low protein conventional meat diet was considered biased, thus, the values demonstrated in the study were not significant [19]. Only two studies [17, 18] were assessed, having a low overall risk of bias, and these constituted the basis for the conclusions. There was a significant difference between the lacto-ovo-vegetarian diet (VD) and the Mediterranean diet (MD) with a mean difference of 4.2 mL/min/1.73m2 (P < 0.001) in final eGFR. However, considering the eGFR at baseline (0.5 ml/min/1.73m2), the relative final difference is 4.7 ml/min/1.73m2. An increase of 3.4 ml/min/1.73 m² was observed between the initial and final eGFR in the VD group, indicating a positive effect of the diet on eGFR (17). In contrast, among patients following the Mediterranean diet, a decrease in glomerular filtration rate of 1.3 ml/min/1.73m2 was observed. It should be noted that in this study the vegetarian diet was compared to the Mediterranean diet. The Mediterranean diet is high in vegetables and low in protein. It seems promising to conduct a study that will compare the vegetarian diet with the usual diet. In the second study, the authors compared a very low protein (KD) vegetarian diet supplemented with ketoanalogs with a standard low protein (LPD) diet. Glomerular filtration rate decreased in both groups. The KD diet caused a smaller reduction in eGFR than LPD, the difference in eGFR decrease was 4.2 ml/min/1.73 m². Although the eGFR was lower at the end of the study in both study groups, the KD diet helped minimize the decline in filtration function (18).

The available scientific sources provide data highlighting other benefits of a vegetarian diet in patients with chronic kidney disease. The crucial aspect is a lower systolic pressure observed in vegetarians, which may have a protective effect not only in terms of the kidneys, but also for the heart. Another vital factor is an anti-inflammatory effects of a plant based diet, which may reduce oxidative stress, protecting against renal injury [22]. Vegetarian diet provides a diversity of a gut microbiome preventing from dysbiosis and low grade inflammation. It can be achieved by the high fiber and vitamin content which increase the bowel transit, preventing from the production of uremic toxins and of reactive oxygen species (ROS) [23]. Vegetarian diet was found to have an anti-inflammatory effect through higher consumption of fruits and vegetables which contain more antioxidative vitamins such as vitamin C, E and beta-carotene [24]. Vegetarians have higher plasma ascorbic acid as well as lower concentration of uric acid and hsCRP [25]. Thanks to this, they have lower risk of developing cardiovascular disease or stroke. Moreover, vegetarians have been found to have lower HO-1 (heme-oxygenase-1) – a marker indicating protective properties against oxidative stress [26]. That constitutes a significant factors in view of the cardiovascular health and the atherosclerosis risk in renal patients. Hyperkalemia, in turn, appears to be a limitation for the prevention of CKD in this group of patients. However, the aforementioned risk is low in patients in stage 4 of CKD, therefore, a vegetarian diet constitutes a safe option for this group [10]. Recent research on the population of CKD patients in stage 4–5 indicated that effects of a very low-protein diet supplemented with ketoanalogues (sVLPD) seem to be as effective as a standard low-protein diet (LPD), with no significant difference in the risk of renal death (P = 0.28) end stage renal disease (ESRD) (P = 0.51), or cardiovascular events (P = 0.2). This provides a new perspective on the idea of the ketoanalogue use in delaying the progression of CKD [27]. In addition to its positive impact on the physical health, a vegetarian diet is considered to positively affect the quality of life (QoL), which comprises several domains, such as physical, social, environmental and psychological. In each of these, vegetarianism plays a vital role in influencing well-being [12]. Although a vegetarian diet seems beneficial, the treatment should be consulted with the dietician to avoid a potential nutritional deficiency.

Although not many RCTs were found, an interesting cohort trial including patients diagnosed with diabetes is worth mentioning. According to a multivariable logistic regression analysis of a cross-sectional study, vegan and ovo-lacto vegetarian diets were found to be less associated with CKD (vegan 0.87, 0.75 to 0.97, P = 0.018; ovo-lacto vegetarian: 0.84, 0.77–0.88, P < 0.001) [28]. Among the patients with a diagnosed T2DM, the risk of CKD was even lower (OR: 0.68, 95% (CI): 0.57– 0.82) in a lacto-ovo vegetarian group as well as (OR 0.68, 95% CI: 0.49–0.94) in the vegan subjects [29]. Moreover, proteinuria was more frequent in the control group (27.7 vs. 21.7% as compared to vegans, and 20.5% in a lacto-ovo vegetarian group, P < 0.001) [29].

### Strengths and limitations

This systematic review bears certain limitations. Only papers in English were included in the review, no publications translated into English, or studies published in other languages were considered in the review process, which may lead to language bias. The availability of relevant studies was also a major limitation of this systematic review, as only four studies met the inclusion criteria. The studies analyzed were found to be heterogeneous in various aspects. There were clinical and methodological differences between studies, such as different inclusion criteria, variability in results due to administered medications, and most significantly - different study designs which may have affected the study results. Another factor was the changing risk of bias. Furthermore, the methods of measuring GFR varied between the studies. In terms of the strengths of this systematic review, the risk of bias was assessed using ROB2 tool in the case of each study.

## Conclusions

As the presented systematic review shows, a vegetarian diet might improve renal filtration function in patients with CKD also delaying its progression. Therefore, given the potential positive effects of a vegetarian diet on the progression of CKD, the patients with CKD should consider following a vegetarian diet in order to improve the renal functions. However, only a small number of studies were eligible and, out of these, only two were considered valuable due to their low bias. Clearly, more research with regard to this topic is necessary, including studies of extended duration, as well as involving larger groups and improved homogeneity. Additionally, it is worth noting other positive aspects of a vegetarian diet, including lower proteinuria and serum urea, which were not explored in this review.

## Electronic supplementary material

Below is the link to the electronic supplementary material.

Supplementary Material 1

Supplementary Material 2

Supplementary Material 3

Supplementary Material 4

Supplementary Material 5

## Data Availability

All data generated or analyzed during this study are included in this paper (and its supplementary information files).

## Abbreviations

BMI: Body mass index
BP: Blood pressure
CKD: Chronic kidney disease
FGF23: Fibroblast growth factor 23
GFR: Glomerular filtration rate
HDL: High-density lipoprotein
HOMA-IR: Homeostatic Model Assessment – Insulin Resistance
LDL: Low-density lipoprotein
TG: Triglycerides
T2DM: Type 2 diabetes mellitus
