# Supplementary material for: The impact of a vegetarian diet on chronic kidney disease (CKD) progression – a systematic review
Source: BMC Nephrol. 2023 Jun 12;24:168. doi: 10.1186/s12882-023-03233-y (PMC10259031; doi:10.1186/s12882-023-03233-y)
Supplement: Supplementary file 1 — Supplementary Material 1 [file 12882_2023_3233_MOESM1_ESM.docx]

Revised Cochrane risk-of-bias tool for randomized crossover trials

TEMPLATE FOR COMPLETION

**Version of 18 March 2021**

The development of the RoB 2 tool was supported by the MRC Network of Hubs for Trials Methodology Research (MR/L004933/2- N61), with the support of the host MRC ConDuCT-II Hub (Collaboration and innovation for Difficult and Complex randomised controlled Trials In Invasive procedures - MR/K025643/1), by MRC research grant MR/M025209/1, and by a grant from The Cochrane Collaboration.


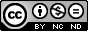


This work is licensed under a [Creative Commons Attribution-NonCommercial-NoDerivatives 4.0 International License](http://creativecommons.org/licenses/by-nc-nd/4.0/).

| **Study details**   \| **Reference** \| Soroka N, Silverberg DS, Greemland M, Birk Y, Blum M, Peer G, et al. Comparison of a vegetable-based (soya) and an animal-based low-protein diet in predialysis chronic renal failure patients. Nephron. 1998;79(2):173–80. \| \| --- \| --- \|   **Study design**   \| □ \| Individually-randomized parallel-group trial \| \| --- \| --- \| \| □ \| Cluster-randomized parallel-group trial \| \| X \| Individually randomized cross-over (or other matched) trial \|   **For the purposes of this assessment, the interventions being compared are defined as**   \| Experimental: \| , soya-based vegetable low-protein diets (VPD) \| Comparator: \| animal-based low-protein diets (APD) \| \| --- \| --- \| --- \| --- \|  \| **Specify which outcome is being assessed for risk of bias** \| eGFR \| \| --- \| --- \|  \| **Specify the numerical result being assessed.** In case of multiple alternative analyses being presented, specify the numeric result (e.g. RR = 1.52 (95% CI 0.83 to 2.77) and/or a reference (e.g. to a table, figure or paragraph) that uniquely defines the result being assessed. \| The GFR was evaluated by Cr-EDTA-measured glomerular  filtration rate ml/min/1.73 m2) Table \| \| --- \| --- \|   **Is the review team’s aim for this result…?**   \| □ \| to assess the effect of *assignment to intervention* (the ‘intention-to-treat’ effect) \| \| --- \| --- \| \| 🞬 \| to assess the effect of *adhering to intervention* (the ‘per-protocol’ effect) \|   **If the aim is to assess the effect of *adhering to intervention***, select the deviations from intended intervention that should be addressed (at least one must be checked):  🞬 occurrence of non-protocol interventions  🞬 failures in implementing the intervention that could have affected the outcome  🞬 non-adherence to their assigned intervention by trial participants  **Which of the following sources were obtained to help inform the risk-of-bias assessment? (tick as many as apply)**  🞬 Journal article(s) with results of the trial  □ Trial protocol  □ Statistical analysis plan (SAP)  □ Non-commercial trial registry record (e.g. ClinicalTrials.gov record)  □ Company-owned trial registry record (e.g. GSK Clinical Study Register record)  □ “Grey literature” (e.g. unpublished thesis)  □ Conference abstract(s) about the trial  □ Regulatory document (e.g. Clinical Study Report, Drug Approval Package)  □ Research ethics application  □ Grant database summary (e.g. NIH RePORTER or Research Councils UK Gateway to Research)  □ Personal communication with trialist  □ Personal communication with the sponsor |
| --- | --- | --- | --- | --- | --- | --- | --- | --- | --- | --- | --- | --- | --- | --- | --- | --- | --- | --- | --- | --- |

## Risk of bias assessment

Responses underlined in green are potential markers for low risk of bias, and responses in red are potential markers for a risk of bias. Where questions relate only to sign posts to other questions, no formatting is used.

**Domain 1a: Risk of bias arising from the randomization process**

| **Signalling questions** | **Comments** | **Response options** |
| --- | --- | --- |
| **1.1 Was the allocation sequence random?** | “The patients were randomly assigned to either group 1 (VPD) or group 2 (APD)” we might assume the random allocation  No information about that, | Y / PY / PN / N / NI |
| **1.2 Was the allocation sequence concealed until participants were enrolled and assigned to interventions?** |  | Y / PY / PN / N / NI |
| **1.3 Did baseline differences between intervention groups at the start of the first period suggest a problem with the randomization process?** | there is a substantial difference in allocation ratio. “Six started on VPD and 3 on APD” it leads to the ratio 2:1. No table presenting the baseline parameters for interventional groups at the beginning | Y / PY / PN / N / NI |
| **Risk-of-bias judgement** |  | Low / High / Some concerns |
| Optional: What is the predicted direction of bias arising from the randomization process? |  | NA / Favours experimental / Favours comparator / Towards null /Away from null / Unpredictable |

**Domain S: Risk of bias arising from period and carryover effects**

| **Signalling questions** | **Comments** | **Response options** |
| --- | --- | --- |
| **S.1 Was the number of participants allocated to each of the two sequences equal or nearly equal?** | ‘Six started on VPD and 3 on APD’ meaning 2:1 ratio | Y/PY/PN/N/NI |
| **S.2 If N/PN/NI to S.1: Were period effects accounted for in the analysis?** | Probably both periods were included | NA/Y/PY/PN/N/NI |
| **S.3 Was there sufficient time for any carryover effects to have disappeared before outcome assessment in the second period?** | ‘They stayed on one diet for 6 months and then switched to the other diet  for a second 6-month period’ no sufficient time for any carryover effects to have disappeared | Y/PY/PN/N/NI |
| **Risk-of-bias judgement** |  | Low / High / Some concerns |
| Optional: What is the predicted direction of bias arising from period and carryover effects? |  | NA / Favours experimental / Favours comparator / Towards null /Away from null / Unpredictable |

**Domain 2: Risk of bias due to deviations from the intended interventions (effect of assignment to intervention)**

| **Signalling questions** | **Comments** | **Response options** |
| --- | --- | --- |
| **2.1. Were participants aware of their assigned intervention during each period of the trial?** | Chosen “assess the effect of adhering to intervention” option | Y / PY / PN / N / NI |
| **2.2. Were carers and people delivering the interventions aware of participants' assigned intervention during each period of the trial?** |  | Y / PY / PN / N / NI |
| **2.3. If Y/PY/NI to 2.1 or 2.2: Were there deviations from the intended intervention that arose because of the trial context?** | Chosen “assess the effect of adhering to intervention” option | NA / Y / PY / PN / N / NI |
| **2.4 If Y/PY to 2.3: Were these deviations likely to have affected the outcome?** | Chosen “assess the effect of adhering to intervention” option | NA / Y / PY / PN / N / NI |
| **2.5. If Y/PY/NI to 2.4: Were these deviations from intended intervention balanced between groups?** | Chosen “assess the effect of adhering to intervention” option | NA / Y / PY / PN / N / NI |
| **2.6 Was an appropriate analysis used to estimate the effect of assignment to intervention?** | Chosen “assess the effect of adhering to intervention” option | Y / PY / PN / N / NI |
| **2.7 If N/PN/NI to 2.6: Was there potential for a substantial impact (on the result) of the failure to analyse participants in the group to which they were randomized?** | Chosen “assess the effect of adhering to intervention” option | NA / Y / PY / PN / N / NI |
| **Risk-of-bias judgement** |  | Low / High / Some concerns |
| Optional: What is the predicted direction of bias due to deviations from intended interventions? |  | NA / Favours experimental / Favours comparator / Towards null /Away from null / Unpredictable |

**Domain 2: Risk of bias due to deviations from the intended interventions (effect of adhering to intervention)**

| **Signalling questions** | **Comments** | **Response options** |
| --- | --- | --- |
| **2.1. Were participants aware of their assigned intervention during each period of the trial?** | Yes they were aware, because there was a change to their diet  No information about that | Y / PY / PN / N / NI |
| **2.2. Were carers and people delivering the interventions aware of participants' assigned intervention during each period of the trial?** |  | Y / PY / PN / N / NI |
| **2.3. [If applicable:] If Y/PY/NI to 2.1 or 2.2: Were important non-protocol interventions balanced between interventions?** | No information about the important non-protocol interventions during the study | NA / Y / PY / PN / N / NI |
| **2.4. [If applicable:] Were there failures in implementing the intervention that could have affected the outcome?** | “Although 15 patients started the study, 6 dropped out: 2 because of cerebrovascular accidents, 1 because sherefused to have the 51Cr-EDTA test performed a second time, 1 because of rapid worsening of the renal function  after receiving an angiotensin-converting enzyme inhibitor in another medical clinic, and 2 because they could not comply with the diet” | NA / Y / PY / PN / N / NI |
| **2.5. [If applicable:] Was there non-adherence to the assigned intervention regimen that could have affected participants’ outcomes?** | “The second patient had no complaints about the diet, but the dietician found that the patient was eating a moderate amount of animal protein as well.” | NA / Y / PY / PN / N / NI |
| **2.6. If N/PN/NI to 2.3, or Y/PY/NI to 2.4 or 2.5: Was an appropriate analysis used to estimate the effect of adhering to the intervention?** | Compliance with the diets was measured in two ways: (1) by a  dietary questionnaire filled out by the dietician at each monthly visit  and (2) by a 24-hour urine sample collected for urea nitrogen and  creatinine determinations every 2 months. The questionnaire, filled  out by the dietician, contained a detailed history of all the patients’  daily intake. | NA / Y / PY / PN / N / NI |
| **Risk-of-bias judgement** |  | Low / High / Some concerns |
| Optional: What is the predicted direction of bias due to deviations from intended interventions? |  | NA / Favours experimental / Favours comparator / Towards null /Away from null / Unpredictable |

**Domain 3: Risk of bias due to missing outcome data**

| **Signalling questions** | **Comments** | **Response options** |
| --- | --- | --- |
| **3.1 Were data for this outcome available for all, or nearly all, participants randomized?** | No 15 patients started the study, 6 dropped out, it means that almost 40% of patients didn’t finish the study | Y / PY / PN / N / NI |
| **3.2 If N/PN/NI to 3.1: Is there evidence that the result was not biased by missing outcome data?** | No evidence that the outcome was not biased | NA / Y / PY / PN / N |
| **3.3 If N/PN to 3.2 Could missingness in the outcome depend on its true value?** | There was a missing outcome data in both groups, GFR has not been accessed due excluded patients. These patients were excluded due to specified reasons | NA / Y / PY / PN / N / NI |
| **3.4 If Y/PY/NI to 3.3: Is it likely that missingness in the outcome depended on its true value?** |  | NA / Y / PY / PN / N / NI |
| **Risk-of-bias judgement** |  | Low / High / Some concerns |
| Optional: What is the predicted direction of bias due to missing outcome data? |  | NA / Favours experimental / Favours comparator / Towards null /Away from null / Unpredictable |

**Domain 4: Risk of bias in measurement of the outcome**

| **Signalling questions** | **Comments** | **Response options** |
| --- | --- | --- |
| **4.1 Was the method of measuring the outcome inappropriate?** | The measuring method was appropriate for this outcome, best laboratory methods were used “Glomerular Filtration Rate (GFR). The GFR was evaluated by(1) 51Cr-EDTA [16] which was measured at the beginning of thestudy, at 6 months, and at 12 months; (2) CCr which was measured every 2 months, and (3) serum creatinine which was measured every 2 months.” | Y / PY / PN / N / NI |
| **4.2 Could measurement or ascertainment of the outcome have differed between interventions within each sequence?** | There was no reported measurement or ascertainment differences between groups and sequences “All patients had been followed up in our outpatient nephrology clinic for a period of at least 1 year” | Y / PY / PN / N / NI |
| **4.3 If N/PN/NI to 4.1 and 4.2: Were outcome assessors aware of the intervention received by study participants?** | No information about that, | NA / Y / PY / PN / N / NI |
| **4.4 If Y/PY/NI to 4.3: Could assessment of the outcome have been influenced by knowledge of intervention received?** | No, because the outcome results are the laboratory measures such as eGFR so cannot be influenced | NA / Y / PY / PN / N / NI |
| **4.5 If Y/PY/NI to 4.4:** **Is it likely that assessment of the outcome was influenced by knowledge of intervention received?** |  | NA / Y / PY / PN / N / NI |
| **Risk-of-bias judgement** |  | Low / High / Some concerns |
| Optional: What is the predicted direction of bias in measurement of the outcome? |  | NA / Favours experimental / Favours comparator / Towards null /Away from null / Unpredictable |

**Domain 5: Risk of bias in selection of the reported result**

| **Signalling questions** | **Comments** | **Response options** |
| --- | --- | --- |
| **5.1 Were the data that produced this result analysed in accordance with a pre-specified analysis plan that was finalized before unblinded outcome data were available for analysis?** | The results were measured with the method according to a pre-specified analysis plan. No outcome measures or analyses have been omitted added to the results report, no post hoc analysis were performed | Y / PY / PN / N / NI |
| **Is the numerical result being assessed likely to have been selected, on the basis of the results, from...** |  |  |
| **5.2. ... multiple eligible outcome measurements (e.g. scales, definitions, time points) within the outcome domain?** | The measurements are the laboratory results (eGFR) | Y / PY / PN / N / NI |
| **5.3 ... multiple eligible analyses of the data?** | No post hoc analyses were performed, only the analyses of pre-specified parameters, no selected results were published | Y / PY / PN / N / NI |
| **5.4 Is a result based on data from both periods sought, but unavailable on the basis of carryover having been identified?** | data from both periods contribute to the result being assessed | Y / PY / PN / N / NI |
| **Risk-of-bias judgement** |  | Low / High / Some concerns |
| Optional: What is the predicted direction of bias due to selection of the reported result? |  | NA / Favours experimental / Favours comparator / Towards null /Away from null / Unpredictable |

Overall risk of bias

| **Risk-of-bias judgement** |  | Low / High / Some concerns |
| --- | --- | --- |
| Optional: What is the overall predicted direction of bias for this outcome? |  | NA / Favours experimental / Favours comparator / Towards null /Away from null / Unpredictable |


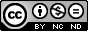


This work is licensed under a [Creative Commons Attribution-NonCommercial-NoDerivatives 4.0 International License](http://creativecommons.org/licenses/by-nc-nd/4.0/).
