# Supplementary material for: The impact of a vegetarian diet on chronic kidney disease (CKD) progression – a systematic review
Source: BMC Nephrol. 2023 Jun 12;24:168. doi: 10.1186/s12882-023-03233-y (PMC10259031; doi:10.1186/s12882-023-03233-y)
Supplement: Supplementary file 2 — Supplementary Material 2 [file 12882_2023_3233_MOESM2_ESM.docx]

Revised Cochrane risk-of-bias tool for randomized crossover trials

TEMPLATE FOR COMPLETION

**Version of 18 March 2021**

The development of the RoB 2 tool was supported by the MRC Network of Hubs for Trials Methodology Research (MR/L004933/2- N61), with the support of the host MRC ConDuCT-II Hub (Collaboration and innovation for Difficult and Complex randomised controlled Trials In Invasive procedures - MR/K025643/1), by MRC research grant MR/M025209/1, and by a grant from The Cochrane Collaboration.


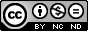


This work is licensed under a [Creative Commons Attribution-NonCommercial-NoDerivatives 4.0 International License](http://creativecommons.org/licenses/by-nc-nd/4.0/).

| **Study details**   \| **Reference** \| de Mello VDF, Zelmanovitz T, Perassolo MS, Azevedo MJ, Gross JL. Withdrawal of red meat from the usual diet reduces albuminuria and improves serum fatty acid profile in type 2 diabetes patients with macroalbuminuria. The American journal of clinical nutrition. 2006 May;83(5):1032–8. \| \| --- \| --- \|   **Study design**   \| □ \| Individually-randomized parallel-group trial \| \| --- \| --- \| \| □ \| Cluster-randomized parallel-group trial \| \| X \| Individually randomized cross-over (or other matched) trial \|   **For the purposes of this assessment, the interventions being compared are defined as**   \| Experimental: \| chicken (CD), lactovegetarian low-protein diet (LPD) \| Comparator: \| red meat in the usual diet (UD) \| \| --- \| --- \| --- \| --- \|  \| **Specify which outcome is being assessed for risk of bias** \| eGFR \| \| --- \| --- \|  \| **Specify the numerical result being assessed.** In case of multiple alternative analyses being presented, specify the numeric result (e.g. RR = 1.52 (95% CI 0.83 to 2.77) and/or a reference (e.g. to a table, figure or paragraph) that uniquely defines the result being assessed. \| Difference of eGFR (mL/min/1.73 m2) between three groups. The results presented in Table 2 \| \| --- \| --- \|   **Is the review team’s aim for this result…?**   \| □ \| to assess the effect of *assignment to intervention* (the ‘intention-to-treat’ effect) \| \| --- \| --- \| \| 🞬 \| to assess the effect of *adhering to intervention* (the ‘per-protocol’ effect) \|   **If the aim is to assess the effect of *adhering to intervention***, select the deviations from intended intervention that should be addressed (at least one must be checked):  🞬 occurrence of non-protocol interventions  🞬 failures in implementing the intervention that could have affected the outcome  🞬 non-adherence to their assigned intervention by trial participants  **Which of the following sources were obtained to help inform the risk-of-bias assessment? (tick as many as apply)**  🞬 Journal article(s) with results of the trial  □ Trial protocol  □ Statistical analysis plan (SAP)  □ Non-commercial trial registry record (e.g. ClinicalTrials.gov record)  □ Company-owned trial registry record (e.g. GSK Clinical Study Register record)  □ “Grey literature” (e.g. unpublished thesis)  □ Conference abstract(s) about the trial  □ Regulatory document (e.g. Clinical Study Report, Drug Approval Package)  □ Research ethics application  □ Grant database summary (e.g. NIH RePORTER or Research Councils UK Gateway to Research)  □ Personal communication with trialist  □ Personal communication with the sponsor |
| --- | --- | --- | --- | --- | --- | --- | --- | --- | --- | --- | --- | --- | --- | --- | --- | --- | --- | --- | --- | --- |

## Risk of bias assessment

Responses underlined in green are potential markers for low risk of bias, and responses in red are potential markers for a risk of bias. Where questions relate only to sign posts to other questions, no formatting is used.

**Domain 1a: Risk of bias arising from the randomization process**

| **Signalling questions** | **Comments** | **Response options** |
| --- | --- | --- |
| **1.1 Was the allocation sequence random?** | ‘patients were randomly assigned to one of the sequences of the intervention diets’ but no stated number of participants allocated to each of three groups.  No data that allocation ratio is 1:1, | Y / PY / PN / N / NI |
| **1.2 Was the allocation sequence concealed until participants were enrolled and assigned to interventions?** |  | Y / PY / PN / N / NI |
| **1.3 Did baseline differences between intervention groups at the start of the first period suggest a problem with the randomization process?** | No data about the baseline differences between intervention groups at the start, there is no useful baseline information available | Y / PY / PN / N / NI |
| **Risk-of-bias judgement** | There are some concerns to random allocation sequence, no data about the intial groups | Low / High / Some concerns |
| Optional: What is the predicted direction of bias arising from the randomization process? |  | NA / Favours experimental / Favours comparator / Towards null /Away from null / Unpredictable |

**Domain S: Risk of bias arising from period and carryover effects**

| **Signalling questions** | **Comments** | **Response options** |
| --- | --- | --- |
| **S.1 Was the number of participants allocated to each of the two sequences equal or nearly equal?** | no stated number of participants allocated to each of three groups | Y/PY/PN/N/NI |
| **S.2 If N/PN/NI to S.1: Were period effects accounted for in the analysis?** | There is no design and analysis strategies that can overcome the potential impact of period effects. Data from the both periods contribute to the result  being assessed for risk of bias. – so no | NA/Y/PY/PN/N/NI |
| **S.3 Was there sufficient time for any carryover effects to have disappeared before outcome assessment in the second period?** | ‘Each diet was followed for 4 wk with a 4-wk washout period between them. During the washout period, the patients maintained their UD’ and in limitation ’ possible carry-over effect probably did not occur because the diets had a 4-wk washout’ | Y/PY/PN/N/NI |
| **Risk-of-bias judgement** |  | Low / High / Some concerns |
| Optional: What is the predicted direction of bias arising from period and carryover effects? | Washout period is a comparator diet | NA / Favours experimental / Favours comparator / Towards null /Away from null / Unpredictable |

**Domain 2: Risk of bias due to deviations from the intended interventions (effect of assignment to intervention)**

| **Signalling questions** | **Comments** | **Response options** |
| --- | --- | --- |
| **2.1. Were participants aware of their assigned intervention during each period of the trial?** | Chosen “assess the effect of adhering to intervention” option | Y / PY / PN / N / NI |
| **2.2. Were carers and people delivering the interventions aware of participants' assigned intervention during each period of the trial?** |  | Y / PY / PN / N / NI |
| **2.3. If Y/PY/NI to 2.1 or 2.2: Were there deviations from the intended intervention that arose because of the trial context?** | Chosen “assess the effect of adhering to intervention” option | NA / Y / PY / PN / N / NI |
| **2.4 If Y/PY to 2.3: Were these deviations likely to have affected the outcome?** | Chosen “assess the effect of adhering to intervention” option | NA / Y / PY / PN / N / NI |
| **2.5. If Y/PY/NI to 2.4: Were these deviations from intended intervention balanced between groups?** | Chosen “assess the effect of adhering to intervention” option | NA / Y / PY / PN / N / NI |
| **2.6 Was an appropriate analysis used to estimate the effect of assignment to intervention?** | Chosen “assess the effect of adhering to intervention” option | Y / PY / PN / N / NI |
| **2.7 If N/PN/NI to 2.6: Was there potential for a substantial impact (on the result) of the failure to analyse participants in the group to which they were randomized?** | Chosen “assess the effect of adhering to intervention” option | NA / Y / PY / PN / N / NI |
| **Risk-of-bias judgement** |  | Low / High / Some concerns |
| Optional: What is the predicted direction of bias due to deviations from intended interventions? |  | NA / Favours experimental / Favours comparator / Towards null /Away from null / Unpredictable |

**Domain 2: Risk of bias due to deviations from the intended interventions (effect of adhering to intervention)**

| **Signalling questions** | **Comments** | **Response options** |
| --- | --- | --- |
| **2.1. Were participants aware of their assigned intervention during each period of the trial?** | They must have known, because it was a trial about the diet  No information about the blinding of people accessing results | Y / PY / PN / N / NI |
| **2.2. Were carers and people delivering the interventions aware of participants' assigned intervention during each period of the trial?** |  | Y / PY / PN / N / NI |
| **2.3. [If applicable:] If Y/PY/NI to 2.1 or 2.2: Were important non-protocol interventions balanced between interventions?** | No non-protocol interventions that trial participants might receive were reported | NA / Y / PY / PN / N / NI |
| **2.4. [If applicable:] Were there failures in implementing the intervention that could have affected the outcome?** | “Compliance with the diets was probably adequate, because its assessment by the weighed diet records method and urea measurements performed during each diet showed a good correlation between these 2 tools…” also the authors reported no failures in implementing the intervention | NA / Y / PY / PN / N / NI |
| **2.5. [If applicable:] Was there non-adherence to the assigned intervention regimen that could have affected participants’ outcomes?** | Not reported | NA / Y / PY / PN / N / NI |
| **2.6. If N/PN/NI to 2.3, or Y/PY/NI to 2.4 or 2.5: Was an appropriate analysis used to estimate the effect of adhering to the intervention?** | No analysis on the effect of adhering to the intervention including ‘Intention to treat (ITT) analysis’, ‘per protocol analysis’, ‘as-treated analysis’, (iv) ‘analysis by treatment | NA / Y / PY / PN / N / NI |
| **Risk-of-bias judgement** |  | Low / High / Some concerns |
| Optional: What is the predicted direction of bias due to deviations from intended interventions? |  | NA / Favours experimental / Favours comparator / Towards null /Away from null / Unpredictable |

**Domain 3: Risk of bias due to missing outcome data**

| **Signalling questions** | **Comments** | **Response options** |
| --- | --- | --- |
| **3.1 Were data for this outcome available for all, or nearly all, participants randomized?** | “All randomly assigned patients completed the study protocol and were included in the final analysis” the authors report that all participants outcomes were available | Y / PY / PN / N / NI |
| **3.2 If N/PN/NI to 3.1: Is there evidence that the result was not biased by missing outcome data?** | Not applicable | NA / Y / PY / PN / N |
| **3.3 If N/PN to 3.2 Could missingness in the outcome depend on its true value?** | Not applicable | NA / Y / PY / PN / N / NI |
| **3.4 If Y/PY/NI to 3.3: Is it likely that missingness in the outcome depended on its true value?** |  | NA / Y / PY / PN / N / NI |
| **Risk-of-bias judgement** |  | Low / High / Some concerns |
| Optional: What is the predicted direction of bias due to missing outcome data? |  | NA / Favours experimental / Favours comparator / Towards null /Away from null / Unpredictable |

**Domain 4: Risk of bias in measurement of the outcome**

| **Signalling questions** | **Comments** | **Response options** |
| --- | --- | --- |
| **4.1 Was the method of measuring the outcome inappropriate?** | The laboratory method is well described without any missing data. There was also an analysis of macronutrients and micronutrients received by the patients “GFR was measured by using the 51Cr-EDTA single-injection technique (CV: 12%; GFR reference range: 72–137.5 mL · min−1 · 1.73 m−2)” – study. The outcome was measured according to laboratory standars | Y / PY / PN / N / NI |
| **4.2 Could measurement or ascertainment of the outcome have differed between interventions within each sequence?** | “At the end of each diet, renal function (UAER and GFR), serum FA, lipid profile, glycemic and anthropometric indexes, and blood pressure were measured” there is also mentioned | Y / PY / PN / N / NI |
| **4.3 If N/PN/NI to 4.1 and 4.2: Were outcome assessors aware of the intervention received by study participants?** | No information about that | NA / Y / PY / PN / N / NI |
| **4.4 If Y/PY/NI to 4.3: Could assessment of the outcome have been influenced by knowledge of intervention received?** | No, because the laboratory result were assessed  Not applicable | NA / Y / PY / PN / N / NI |
| **4.5 If Y/PY/NI to 4.4:** **Is it likely that assessment of the outcome was influenced by knowledge of intervention received?** |  | NA / Y / PY / PN / N / NI |
| **Risk-of-bias judgement** |  | Low / High / Some concerns |
| Optional: What is the predicted direction of bias in measurement of the outcome? |  | NA / Favours experimental / Favours comparator / Towards null /Away from null / Unpredictable |

**Domain 5: Risk of bias in selection of the reported result**

| **Signalling questions** | **Comments** | **Response options** |
| --- | --- | --- |
| **5.1 Were the data that produced this result analysed in accordance with a pre-specified analysis plan that was finalized before unblinded outcome data were available for analysis?** | Yes the data were collected according to pre specified plan | Y / PY / PN / N / NI |
| **Is the numerical result being assessed likely to have been selected, on the basis of the results, from...** |  |  |
| **5.2. ... multiple eligible outcome measurements (e.g. scales, definitions, time points) within the outcome domain?** | There is only one possible way in which the outcome domain can be measured, it is a eGFR measurements | Y / PY / PN / N / NI |
| **5.3 ... multiple eligible analyses of the data?** | All data is fully reported as previously specified no selection of data has been performed | Y / PY / PN / N / NI |
| **5.4 Is a result based on data from both periods sought, but unavailable on the basis of carryover having been identified?** | data from both periods contribute to the result assessed | Y / PY / PN / N / NI |
| **Risk-of-bias judgement** |  | Low / High / Some concerns |
| Optional: What is the predicted direction of bias due to selection of the reported result? |  | NA / Favours experimental / Favours comparator / Towards null /Away from null / Unpredictable |

Overall risk of bias

| **Risk-of-bias judgement** |  | Low / High / Some concerns |
| --- | --- | --- |
| Optional: What is the overall predicted direction of bias for this outcome? |  | NA / Favours experimental / Favours comparator / Towards null /Away from null / Unpredictable |


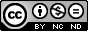


This work is licensed under a [Creative Commons Attribution-NonCommercial-NoDerivatives 4.0 International License](http://creativecommons.org/licenses/by-nc-nd/4.0/).
